# Hidden hospital mortality in patients with sepsis discharged from the intensive care unit

**DOI:** 10.5935/0103-507X.20190037

**Published:** 2019

**Authors:** Inês Aguiar-Ricardo, Hélia Mateus, João Gonçalves-Pereira

**Affiliations:** 1 Departamento de Cardiologia, Hospital Santa Maria - Lisboa, Portugal.; 2 Departamento de Clínica Médica, Hospital Leiria - Portugal.; 3 Departamento de Cuidados Intensivos, Hospital Vila Franca de Xira - Portugal.; 4 Nova Medical School - Lisboa, Portugal.

**Keywords:** Sepsis, Hospital mortality, Intensive care units, Sepse, Mortalidade hospitalar, Unidades de terapia intensiva

## Abstract

**Objective:**

To evaluate the impact of the presence of sepsis on in-hospital mortality after intensive care unit discharge.

**Methods:**

Retrospective, observational, single-center study. All consecutive patients discharged alive from the intensive care unit of *Hospital Vila Franca de Xira* (Portugal) from January 1 to December 31, 2015 (N = 473) were included and followed until death or hospital discharge. In-hospital mortality after intensive care unit discharge was calculated for septic and non-septic patients.

**Results:**

A total of 61 patients (12.9%) died in the hospital after being discharged alive from the intensive care unit. This rate was higher among the patients with sepsis on admission, 21.4%, whereas the in-hospital, post-intensive care unit mortality rate for the remaining patients was nearly half that, 9.3% (p < 0.001). Other patient characteristics associated with mortality were advanced age (p = 0.02), male sex (p < 0.001), lower body mass index (p = 0.02), end-stage renal disease (p = 0.04) and high Simplified Acute Physiology Score II (SAPS II) at intensive care unit admission (p < 0.001), the presence of shock (p < 0.001) and medical admission (p < 0.001). We developed a logistic regression model and identified the independent predictors of in-hospital mortality after intensive care unit discharge.

**Conclusion:**

Admission to the intensive care unit with a sepsis diagnosis is associated with an increased risk of dying in the hospital, not only in the intensive care unit but also after resolution of the acute process and discharge from the intensive care unit.

## Abstract (pt)

**Objetivo:**

Avaliar o impacto da presença de sepse na mortalidade hospitalar após alta da unidade de terapia intensiva.

**Métodos:**

Ensaio retrospectivo, observacional, em centro único. Todos os pacientes que consecutivamente receberam alta vivos da unidade de terapia intensiva do Hospital Vila Franca de Xira (Portugal) entre 1º de janeiro e 31 de dezembro de 2015 (N = 473) foram incluídos e acompanhados até o óbito ou alta do hospital. A mortalidade hospitalar após alta da unidade de terapia intensiva foi calculada para pacientes sépticos e não sépticos.

**Resultados:**

Um total de 61 pacientes (12,9%) faleceu no hospital após receber alta vivos da unidade de terapia intensiva. Esta taxa foi mais elevada entre os pacientes que tinham sepse quando da admissão (21,4%), enquanto a taxa de mortalidade hospitalar após alta da unidade de terapia intensiva para os demais pacientes foi aproximadamente a metade (9,3%), com p < 0,001. Outras características dos pacientes associadas com mortalidade foram idade avançada (p = 0,02), sexo masculino (p < 0,001), índice mais baixo de massa corporal (p = 0,02), nefropatia terminal (p = 0,04) e, quando da admissão à unidade de terapia intensiva, escore elevado segundo o *Simplified Acute Physiology Score* (SAPS II); p < 0,001, presença de choque (p < 0,001) e admissão por causas clínicas (p < 0,001). Desenvolvemos um modelo de regressão logística e identificamos os preditores independentes de mortalidade hospitalar após alta da unidade de terapia intensiva.

**Conclusão:**

A admissão à unidade de terapia intensiva com diagnóstico de sepse se associa com maior risco de morrer no hospital, não apenas na unidade de terapia intensiva quanto também após a resolução do processo agudo e alta da unidade de terapia intensiva.

## INTRODUCTION

Sepsis is a potentially fatal condition associated with organ dysfunction that is caused by a dysregulated host response triggered by an infection.^(1)^ It is a major cause of hospitalization in the intensive care unit (ICU) and is associated with increased morbidity and mortality.^(2)^

In Portugal, due to the small number of available ICU beds,^(3)^ infection and sepsis, which are more readily recognized in severe patients, is highly prevalent, and sepsis patients have a higher mortality rate.^(2)^

It is well known that a significant fraction of patients discharged from the ICU to the ward die in the hospital. In a recent meta-analysis of 58 studies that included more than 2 million patients, 3 to 7% of the ICU patients discharged to the ward died before leaving the hospital.^(4)^ Some of these patients had limitations of care due to the severity of their clinical condition, whether it was acute or chronic.^(5)^ However, a large number died unexpectedly, which seems to be related to both comorbidities and the acute clinical insult.^(2,6)^

The decision to discharge a patient to the ward is often based on clinical criteria, namely, "stabilization", which is often poorly validated. The use of such criteria may be especially important in populations at risk of dying in the ward after ICU discharge. With a careful approach to the data presented by some epidemiological studies,^(2,6)^ it is possible to observe a significant excess of mortality in the ward among patients admitted to the ICU with sepsis. This excess may be related to premature discharge and suboptimal therapeutic approaches and care on the ward.^(7,8)^ Identifying these high-risk populations should be of paramount importance to improve the process of care,^(9)^ namely by creating objective criteria for discharge or for the use of progressive transition.^(10)^

In this study, we evaluated the mortality and hospital length of stay of patients discharged from the ICU without an indication for limitation of care. We measured the impact of the presence of sepsis on admission to the ICU on in-hospital mortality (i.e., mortality that occurred after being discharged alive from the ICU). We also evaluated potential risk factors for in-hospital, post-ICU mortality, focusing on those that were identifiable during hospitalization in the ICU.

## METHODS

We performed a single-center, retrospective, observational study to assess in-hospital mortality after ICU discharge. The *Hospital Vila Franca de Xira* Research and Ethics Committee approved the design of the study. Informed consent was waived due to the observational and retrospective nature of the study.

All consecutive patients discharged alive from the ICU of Hospital Vila Franca de Xira (Portugal) for one year, from January 1 to December 31, 2015, were included. Patients for whom there was a decision to limit of care or to preclude readmission to the ICU (N = 18) were excluded from further analysis.

*Hospital Vila Franca de Xira* is a small hospital near Lisbon (260 beds). As expected, the ICU receives mainly medical and surgical patients (roughly 75% - 25%), with a very low rate of trauma patients. The mean Simplified Acute Physiology Score (SAPS) II score of patients is usually high (46.4 in 2015). The ICU has 8 level III beds. An intermediate care unit with 12 beds is associated with the same department and has common nursing and medical staff.

In this study, we collected demographic data, including sex, age, type of admission (either surgical or medical) and location (emergency room, operating room, ward). Laboratory data on admission (lactate) and clinical information (the presence of shock, comorbidities, such as hypertension, diabetes mellitus, chronic renal disease, heart failure and active neoplasia) were recorded. Disease severity on admission to the ICU was assessed by the calculation of the SAPS II. Sepsis was defined as the presence of infection (suspected or microbiologically documented) associated with systemic manifestations, including organ failure,^(1)^ which led to the provision of antibiotic therapy, according to the physician in charge. The infection was categorized as acquired in the community or in the hospital, according to commonly used criteria.^(1)^ When a patient experienced more than one infectious episode, each was recorded and accounted for. Each patient was included only once (first episode of hospitalization in the ICU).

All data were recorded in a file specially created for this study.

### Statistical analysis

Continuous variables are expressed as the mean ± standard deviation according to their distribution; discrete variables are expressed as the total number (percentage).

Univariate analysis was performed using the chi-square test (for discrete variables) or Student's T or Mann-Whitney tests (for continuous variables) according to the data distribution. The variables that were identified in the univariate analysis as potentially associated with in-hospital mortality after ICU discharge (considering a p < 0.1) were included in a logistic regression model. All these variables were assessed 2 to 2 for the exclusion of significant correlations. A coefficient r < 0.3 was considered sufficient to exclude this correlation. In cases where a correlation was found, the variable considered to be of the greatest clinical importance was included in the model. For the logistic regression model, the results are expressed as odds ratios (OR) with 95% confidence intervals (95%CI).

As the SAPS II score is predictive of in-ward mortality, and we believe that the inclusion of this score can compensate for possible imbalances that may exist in patients' admission characteristics. In fact, the SAPS II score accounts for age, type of admission (either surgical or medical), presence of significant comorbidities and clinical severity on admission to the ICU.

All statistical tests were two-tailed, and a value of p < 0.05 was considered statistically significant. Statistical analysis was performed using the Statistical Package for Social Science (SPSS) version21 (SAS Institute, Cary, NC) software package.

## RESULTS

During the study period, 473 patients were discharged alive from the ICU without a decision to limit care or to preclude ICU readmission. All of these patients were retrospectively screened for the presence of infection and sepsis at ICU admission (N = 140, 29.6%).

The clinical characteristics of patients with or without infection upon admission to the ICU are presented in table 1.

**Table 1:** Clinical characteristics at admission of patients discharged alive from the intensive care unit, according to the presence of infection

	No sepsis (N = 333)	Sepsis (N = 140)	p value
Age	67.3 ± 14.3	65.9 ± 16	0.35*
Male sex	193 (58)	72 (51.4)	0.22†
BMI	28.7 ± 6.7	27.3 ± 5.7	0.03*
Type of admission			
Medical	240 (72.1)	109 (77.9)	
Elective surgery	36 (10.8)	3 (2.1)	
Urgent surgery	41 (12.3)	28 (20)	
Trauma	16 (4.8)	0 (0)	< 0.001†
Comorbidities			
Diabetes mellitus	115 (34.6)	42 (30)	0.34†
Active cancer	45 (13.5)	16 (11.4)	0.65†
End-stage renal disease	13 (3.9)	8 (5.7)	0.46†
Chronic heart failure	93 (27.9)	28 (20)	0.08†
Arterial hypertension	199 (59.8)	75 (53.6)	0.22
Severity of disease			
Shock	48 (14.4)	42 (30)	< 0.001†
ICU-acquired infection	16 (4.8)	21 (15)	0.001†
SAPS II score	37.4 ± 16.1	42.5 ± 17	0.03*
Lactate	1.7 [1.2 - 2.8]	1.9 [1.4 - 2.9]	0.17‡
Glasgow Coma Score	13.5 ± 2.7	13.6 ± 2.5	0.67*
Length of ICU stay	3 [2 - 4]	3 [2 - 7]	< 0.001‡
In-hospital mortality	31 (9.3)	30 (21.4)	< 0.001‡
Readmission to the ICU	17 (5.1)	8 (5.7)	0.79

BMI - body mass index; ICU - intensive care unit; SAPS - Simplified Acute Physiology Score.

* Student’s t test;

† Chi square test;

‡ Mann-Whitney U test. Data are presented as N (%), mean ± standard deviation or median [interquartile range], according to data distribution.

Infected patients more often had a severe disease on admission (significantly higher SAPS II and the presence of shock). Intensive care unit-acquired infections were also more frequent in these patients (15% *versus* 4.8%, p = 0.001). In contrast, comorbidities were more commonly reported in noninfected patients (Table 1).

After discharge from the ICU, 61 (12.9%) patients died in the hospital, usually on the ward. As expected, the SAPS II was lower in patients who were discharged alive from the hospital (37.1 ± 15.7 *versus* 51.5 ± 16.4, p < 0.001) (Table 2).

**Table 2:** Univariate analysis of factors associated with mortality after intensive care unit discharge

	Survivors (N = 412)	Nonsurvivors (N = 61)	p value
Age	66.2 ± 14.6	71.2 ± 15.5	0.02*
Male sex	223 (54.1)	42 (68.9)	< 0.001†
BMI	28.5 ± 6.5	26.4 ± 6.3	0.02*
Type of admission			
Medical	295 (71.6)	54 (88.5)	
Elective surgery	38 (9.2)	1 (1.6)	
Urgent surgery	64 (15.5)	5 (8.2)	
Trauma	15 (3.6)	1 (1.6)	< 0.001†
Sepsis on admission	110 (26.7)	30 (49.2)	0.001†
ICU-acquired infection	28 (6.8)	9 (14.8)	0.04†
Comorbidities			
Diabetes mellitus	139 (33.8)	18 (29.5)	0.56†
Active cancer	56 (13.6)	5 (8.2)	0.31†
End-stage renal disease	15 (3.6)	6 (9.8)	0.04†
Chronic heart failure	107 (26)	14 (23)	0.75†
Arterial hypertension	244 (59.2)	30 (49.2)	0.17†
Severity of disease			
Shock	62 (15)	28 (45.9)	< 0.001†
SAPS II score	37.1 ± 15.7	51.5 ± 16.4	< 0.001*
Lactate	1.7 [1.2 - 2.6]	2.5 [1.4 - 4.5]	0.09‡
Glasgow Coma Score	13.5 ± 2.7	13.6 ± 2.5	0.67*

BMI - body mass index; ICU - intensive care unit; SAPS - Simplified Acute Physiology Score.

* Student’ T test;

† Chi square test;

‡ Kruskal-Wallis test. Data presented as N (%), mean ± standard deviation or median [interquartile range], according to data distribution.

The patients who died were significantly older (71.2 ± 15.5 *versus* 66.2 ± 14.6; p = 0.02), more frequently (68.9%) were male (p < 0.001), more often were admitted to the ICU for a medical reason (88.5%) and had non -clinically relevant higher median lactate levels on admission (2.5 [1.4 - 4.5] *versus* 1.7 [1.2 - 2.6]; p = 0.09).

Again, the presence of sepsis at admission to the ICU was associated with the risk of dying in the hospital, after ICU discharge (21.1% *versus* 10.7%, p = 0.001), but not with ICU readmission (Table 1). In our cohort, patients discharged after an episode of bacteremia had a higher mortality risk (Table 3). Having had a respiratory or abdominal focus of infection was also associated with an increased risk of dying on the ward.

**Table 3:** Focus of infection of the septic patients and outcomes

	N	Mortality	Age	SAPS II	Male sex
Abdominal	33	7 (21.2)	69.6 ± 14.2	45.6 ± 15.7	12 (36.4)
Bacteremia	8	3 (37.5)	72.9 ± 4.7	38.8 ± 13.0	5 (62.5)
Respiratory	57	15 (26.3)	66.2 ± 17.5	44.2 ± 17.6	33 (57.9)
Skin and soft tissues	6	1 (16.7)	69.0 ± 18.6	37.5 ± 14.8	4 (66.7)
Urinary	20	2 (10)	63.4 ± 13.6	41.8 ± 19.0	5 (25)
CNS	5	0 (0)	56.0 ± 15.4	26.2 ± 20.8	2 (40)
Other	11	2 (18.2)	55.5 ± 18.1	38.5 ± 14.0	11 (100)

SAPS - Simplified Acute Physiology Score; CNS - central nervous system. Data presented as N (%) or mean ± standard deviation.

Interestingly, of the other studied comorbidities, namely, active cancer, HIV infection, chronic heart failure, arterial hypertension or diabetes mellitus, none were associated with in-hospital mortality after discharge from the ICU (Table 2).

Accordingly, we developed a logistic regression model to evaluate the independent associations with the risk of dying in the hospital, after ICU discharge. We found that the presence of sepsis on admission to the ICU (OR 2.32, 95%CI 1.28 - 4.24), male sex (OR 2.26, 95%CI 1.21 - 4.24) and the SAPS II score (OR per point 1.05, 95%CI 1.03 - 1.07) were independent predictors of in-hospital death (Table 4).

**Table 4:** Independent predictors of in-hospital mortality after intensive care unit discharge

	OR	95%CI	p value
Male sex	2.26	1.21 - 4.24	0.012
SAPS II (per point)	1.05	1.03 - 1.07	< 0.001
Sepsis on admission	2.32	1.28 - 4.24	0.006

OR - odds ratio; 95%CI - confidence interval; SAPS - Simplified Acute Physiology Score. Also included in the model: age, lactate, diagnosis of end-stage renal disease, intensive care unit-acquired infection, body mass index, and type of admission. Shock was excluded because of collinearity with the SAPS II.

## DISCUSSION

In this study, we report an association between sepsis on admission to the ICU and hospital mortality that persists after resolution of the acute disease and discharge from the ICU (OR 2.32, 95%CI 1.28 - 4.24) and is independent of comorbidities or age.

This association of sepsis with mortality in the ICU is well known^(6)^ and may persist even after hospital discharge,^(11)^ eventually becoming associated with a high incidence or worsening of a previously existent cardiovascular disease.^(12)^ Although the relationship of cardiovascular disease risk with sepsis decreases with time, it may persist for several years.^(12)^

In this study, we focused only on mortality in the hospital after ICU discharge. We believe that this risk factor, sepsis on admission to the ICU, should be taken into account when allocating resources, namely, in the admission of patients to functional units with greater availability of human resources, the use of progressive step-down or hand-over programs or even the prolongation of hospitalization in the ICU, to help reduce morbidity and mortality. Our data suggests that such measures are especially important for patients admitted with bacteremia or an abdominal or respiratory infection.

Some patients die soon after discharge from the ICU, and most of these deaths are associated with a plan to limit care and are to be expected. In our study, we identified and excluded 18 patients in this situation. However, other patients die unexpectedly on the ward, and these death could potentially be avoided with a better process of care.^(13)^

Several patients are discharged from the ICU to an intermediate care unit to receive a higher standard of care than can be provided on the ward. Nevertheless, a study of 690 patients discharged from the ICU failed to demonstrate an association between ICU discharge to an intermediate care unit and a better outcome.^(14)^ Of note, another multicenter study showed a survival benefit for the overall population when intermediate care units were available in the hospital (adjusted OR 0.63, 95%CI 0.45 to 0.88), although this benefit was not necessarily related to admission to those units.^(15)^

We clearly need to better understand the prognostic factors associated with this late mortality risk to identify patients who may benefit from this resource. In a British study, Daly et al.^(16)^ suggested that prolonging the hospitalization of critically ill patients with sepsis in the ICU for an additional 48 hours could reduce hospital mortality by approximately 39%. In addition, even the hour and weekday of discharge may influence the outcome of these patients,^(17)^ probably in relation to the varying availability of resources in the hospital. Discharge to an intermediate care unit often occurs at late hours,^(18)^ which may jeopardize the potential benefits of a progressive step-down approach.

Lactate has also been proposed as a possible prognostic factor for patients admitted to the ICU,^(19)^ especially the septic population.^(20)^ However, there are multiple pitfalls in the interpretation of either absolute or relative lactate levels,^(21)^ and their relationship with outcomes may not be straightforward, as we found in our study. Another potential prognostic factor is the C-reactive protein concentration at ICU discharge, which may help to identify patients at risk.^(22,23)^

The in-hospital mortality rate of patients discharged from the ICU in our study was high, 12.9%; this was in sharp contrast with a meta-analysis published in 2014, where the mean hospital mortality after ICU discharge was only 6.8%.^(4)^ However, these numbers are common in Portuguese ICUs.^(2,22)^ We believe that these higher values were mostly related to the case mix, namely, the high prevalence of comorbidities in the Portuguese ICU population,^(2)^ the predominance of patients admitted for medical reasons or sepsis and even the high prevalence of patients admitted from the ward.

On the other hand, a higher late mortality on the ward among patients admitted to the ICU with sepsis was evident in other studies, although it was rarely mentioned. In the International Study of Prevalence and Outcomes of Infection in Intensive Care Units (EPIC) II,^(6)^ the in-hospital mortality after ICU discharge was 7.3%. This mortality rate was clearly higher in those patients who were deemed to be infected on the study day: 10.3% in infected patients *versus* 4.6% in others (estimated OR 2.4, p < 0.001).^(6)^ In the Portuguese multicenter study Infection on Admission to the Intensive Care (INFAUCI), the in-hospital mortality after ICU discharge was 11.5%, but it was higher (14.2%) in patients who had an infection on admission (9.6% in the others, OR 1.6, p < 0.001).^(2)^ In another cohort of surgical patients, the mortality risk in the first year after hospital discharge was also increased in the septic population (OR 1.71, 95%CI 1.46 - 2.00), particularly among the elderly (> 75 years) and those with the highest Charlson scores.^(24)^

In fact, this persistent mortality risk among the septic population may be related to the continuation of the inflammatory state that persists even after the clinical resolution of sepsis, a risk that was unveiled in patients addmited with community-acquired pneumonia.^(25)^ In a multicenter study, GenIMS, ^(25)^ high levels of IL-6 at hospital discharge were associated with a higher 3-month mortality that may be related to a high prevalence of new or worsening cardiovascular disease, which may persist for several years.^(12,26)^

Consequently, we believe that a more aggressive approach to care for these patients, not only in the short term but also in the long term, may lead to a reduction in mortality and morbidity.

Our study has several limitations: it was a retrospective, single-center study. Sepsis was identified by the physician in charge (at ICU discharge), and the risk of misclassification was real. Moreover, our exclusion of patients with a limitation of care were based only on the decision made at ICU discharge and did not consider eventual additional decisions made later, on the ward.

We adjusted the mortality risk to the SAPS II score. However, these scores is determined at ICU admission and do not include some important predictors of mortality at ICU discharge, such as the level of consciousness, the presence of tracheostomy, the need for noninvasive ventilation, ICU-acquired muscular weakness, and ulcerations.

However, our study focused specifically on the in-hospital period after ICU discharge (usually "blind" in epidemiological studies), excluded patients with a plan to limit care (who naturally have higher mortality rates and contaminate the interpretation of the results), included consecutive patients throughout a whole year (thus eliminating seasonal variations that may exist) and evaluated data for different comorbidities. Furthermore, the identified mortality was relatively high, which allowed the impact of different risk factors to be discriminated.

## CONCLUSION

In conclusion, we found an association between being admitted to the intensive care unit with an infectious disease and sepsis and an increased risk of dying in the hospital, even after discharge from the intensive care unit after the acute process was resolved. This association was independent of age or comorbidities.

## References

[1] Singer M, Deutschman CS, Seymour CW, Shankar-Hari M, Annane D, Bauer M (2016). The Third International Consensus Definitions for Sepsis and
Septic Shock (Sepsis-3). JAMA.

[2] Gonçalves-Pereira J, Pereira JM, Ribeiro O, Baptista JP, Froes F, Paiva JA (2014). Impact of infection on admission and of the process of care on
mortality of patients admitted to the intensive care unit: the INFAUCI
study. Clin Microbiol Infect.

[3] Rhodes A, Ferdinande P, Flaatten H, Guidet B, Metnitz PG, Moreno RP (2012). The variability of critical care bed numbers in
Europe. Intensive Care Med.

[4] Hosein FS, Roberts DJ, Turin TC, Zygun D, Ghali WA, Stelfox HT (2014). A meta-analysis to derive literature-based benchmarks for
readmission and hospital mortality after patient discharge from intensive
care. Crit Care.

[5] Fernandez R, Bacelar N, Hernandez G, Tubau I, Baigorri F, Gili G (2008). Ward mortality in patients discharged from the ICU with
tracheostomy may depend on patient's vulnerability. Intensive Care Med.

[6] Vincent JL, Rello J, Marshall J, Silva E, Anzueto A, Martin CD, Moreno R, Lipman J, Gomersall C, Sakr Y, Reinhart K, EPIC II Group of Investigators (2009). International study of the prevalence and outcomes of infection
in intensive care units. JAMA.

[7] Wallis CB, Davies HT, Shearer AJ (1997). Why do patients die on general wards after discharge from
intensive care units?. Anaesthesia.

[8] Lawrence A, Havill JH (1999). An audit of deaths occurring in hospital after discharge from the
intensive care unit. Anaesth Intensive Care.

[9] Elliott M, Worrall-Carter L, Page K (2012). Factors associated with in-hospital mortality following ICU
discharge: a comprehensive review. Br J Intensive Care.

[10] Niven DJ, Bastos J, Stelfox HT (2014). Critical care transition programs and the risk of readmission or
death after discharge from an ICU: a systematic review and
meta-analysis. Crit Care Med.

[11] Prescott HC, Osterholzer JJ, Langa KM, Angus DC, Iwashyna TJ (2016). Late mortality after sepsis: propensity matched cohort
study. BMJ.

[12] Bergh C, Fall K, Udumyan R, Sjöqvist H, Fröbert O, Montgomery S (2017). Severe infections and subsequent delayed cardiovascular
disease. Eur J Prev Cardiol.

[13] Abizanda Campos R, Altaba Tena S, Belenguer Muncharaz A, Más Font S, Ferrándiz Sellés A, Mateu Campos L (2011). Study of post-ICU mortality during 4 years (2006-2009). Analysis
of the factors related to death in the ward after discharge from the
ICU. Med intensiva.

[14] Ranzani OT, Zampieri FG, Taniguchi LU, Forte DN, Azevedo LC, Park M (2014). The effects of discharge to an intermediate care unit after a
critical illness: a 5-year cohort study. J Crit Care.

[15] Capuzzo M, Volta C, Tassinati T, Moreno R, Valentin A, Guidet B, Iapichino G, Martin C, Perneger T, Combescure C, Poncet A, Rhodes A, Working Group on Health Economics of the European Society of
Intensive Care Medicine (2014). Hospital mortality of adults admitted to intensive care units in
hospitals with and without intermediate care units: a multicentre European
cohort study. Crit Care.

[16] Daly K, Beale R, Chang RW (2001). Reduction in mortality after inappropriate early discharge from
intensive care unit: logistic regression triage model. BMJ.

[17] Moreira HE, Verga F, Barbato M, Burghi G (2017). Prognostic impact of the time of admission and discharge from the
intensive care unit. Rev Bras Ter Intensiva.

[18] Wunsch H, Harrison DA, Jones A, Rowan K (2014). The impact of the organization of high-dependency care on acute
hospital mortality and patient flow for critically ill
patients. Am J Respir Crit Care Med.

[19] Fuller BM, Dellinger RP (2012). Lactate as a hemodynamic marker in the critically
ill. Curr Opin Crit Care.

[20] Machado Rde L, David CM, Luiz RR, Amitrano Dde A, Salomão Cde S, Oliveira GM (2009). Related prognostic factors in elderly patients with severe sepsis
and septic shock. Rev Bras Ter Intensiva.

[21] Marik PE, Bellomo R (2013). Lactate clearance as a target of therapy in sepsis: a flawed
paradigm. Crit Care.

[22] Araújo I, Goncalves-Pereira J, Teixeira S, Nazareth R, Silvestre J, Mendes V (2012). Assessment of risk factors for in-hospital mortality after
intensive care unit discharge. Biomarkers.

[23] Ranzani OT, Prada LF, Zampieri FG, Battaini LC, Pinaffi JV, Setogute YC (2012). Failure to reduce C-reactive protein levels more than 25% in the
last 24 hours before intensive care unit discharge predicts higher
in-hospital mortality: a cohort study. J Crit Care.

[24] Ou L, Chen J, Hillman K, Flabouris A, Parr M, Assareh H (2017). The impact of post-operative sepsis on mortality after hospital
discharge among elective surgical patients: a population-based cohort
study. Crit Care.

[25] Yende S, D'Angelo G, Kellum JA, Weissfeld L, Fine J, Welch RD, Kong L, Carter M, Angus DC (2008). Inflammatory markers at hospital discharge predict subsequent
mortality after pneumonia and sepsis. Am J Respir Crit Care Med.

[26] Eurich DT, Marrie TJ, Minhas-Sandhu JK, Majumdar SR (2017). Risk of heart failure after community acquired pneumonia:
prospective controlled study with 10 years of follow-up. BMJ.

